# Herpes Zoster Is Associated with Prior Statin Use: A Population-Based Case-Control Study

**DOI:** 10.1371/journal.pone.0111268

**Published:** 2014-10-24

**Authors:** Shiu-Dong Chung, Ming-Chieh Tsai, Shih-Ping Liu, Herng-Ching Lin, Jiunn-Horng Kang

**Affiliations:** 1 Division of Urology, Department of Surgery, Far Eastern Memorial Hospital, New Taipei City, Taiwan; 2 Sleep Research Center, Taipei Medical University Hospital, Taipei, Taiwan; 3 Division of Gastroenterology, Department of Internal Medicine, Cathay General Hospital, Taipei, Taiwan; 4 School of Health Care Administration, Taipei Medical University, Taipei, Taiwan; 5 Department of Urology, National Taiwan University Hospital and College of Medicine, Taipei, Taiwan; 6 Department of Physical Medicine and Rehabilitation, Taipei Medical University Hospital, Taipei, Taiwan; 7 Department of Physical Medicine and Rehabilitation, School of Medicine, College of Medicine, Taipei Medical University, Taipei, Taiwan; University of California Riverside, United States of America

## Abstract

**Background:**

This study investigated the association between statin use and herpes zoster (HZ) occurrence in a population-based case-control study.

**Methods:**

Study subjects were retrieved from the Taiwan Longitudinal Health Insurance Database 2000. This study included 47,359 cases with HZ and 142,077 controls. We performed conditional logistic regression analyses to calculate the odds ratio (OR) to present the association between HZ and having previously been prescribed statin.

**Results:**

We found that 13.0% of the sampled subjects had used statins, at 15.5% and 12.1% for cases and controls, respectively (*p*<0.001). A conditional logistic regression analysis suggested that the adjusted OR of being a statin user before the index date for cases was 1.28 (95% confidence interval (CI): 1.24∼1.32) compared to controls. Subjects aged 18∼44 years had the highest adjusted OR for prior statin use among cases compared to controls (OR: 1.69; 95% CI: 1.45∼1.92). Furthermore, we found that the ORs of being a regular and irregular statin user before the index date for cases were 1.32 (95% CI: 1.27∼1.38) and 1.23 (95% CI: 1.181.29), respectively, compared to controls.

**Conclusions:**

We concluded that prior statin use was associated with HZ occurrence.

## Introduction

Statin is a commonly prescribed drug to lower blood lipid levels and reduce cardiovascular risks via inhibition of the HMG-CoA reductase pathway. It was also found that statin has pleiotropic effects on reducing inflammation, enhancing nitric oxide production, increasing insulin sensitivity, and improving bone density [1]–[3].

Herpes zoster (HZ) manifests as a painful vesicle that spans one or two dermatomes in the body. HZ is caused by reactivation of infection by a latent varicella-zoster virus (VZV) that hibernates in dorsal root ganglia. HZ can still cause significant and prolonged neuralgia, disability, and medical burdens [4]–[6]. Very few previous study suggested an association between statin use and HZ occurrence. Terao et al. performed a case-control study in Japan which showed that statin may increase HZ occurrence [7]. Nevertheless, that study possibly suffered from selection bias with its single hospital-based design. The relatively small sample size may have limited its statistical power. Recently, a large retrospective study form Canada showed that the statin users were associated with increased small risk to have HZ comparing to statin non-users [8]. However, only older people (>66 years-old) were included in that study. In the present study, we explored the association between statin and HZ occurrence in a population-based case-control study in Taiwan mainly consisted with Chinese population. The results of the study are important for elucidating potential adverse effects of statin.

## Methods

### Database

Study subjects were retrieved from the Longitudinal Health Insurance Database (LHID2000), derived from the Taiwan National Health Insurance (NHI) program. The LHID2000 mainly includes the medical claims of 1,000,000 enrollees who were randomly selected from all enrollees (*n* = 23.72 million) listed in the 2000 Registry of Beneficiaries under the NHI program. Taiwan's National Health Research Institute and other researchers have validated the completeness and accuracy of the claims data of NHI research database [9], [10]. The LHID2000, which was open to the researchers in Taiwan, was available from the Taiwan's National Health Research Institute (http://nhird.nhri.org.tw/date_01.html). You can contact nhird@nhri.org.tw for data application. Since the LHID2000 consists of de-identified secondary data released to the public for research purposes, this study was exempted from full review by the Institutional Review Board (IRB) after consulting with the director of the Taipei Medical University IRB.

### Selection of cases and controls

For cases, we first identified all subjects who had visited ambulatory care centers (including outpatient departments of hospitals and clinics) and received a first-time diagnosis of HZ (ICD-9-CM code 053) between 1 January 2001 and 31 December 2011 (*n* = 47,521). The date of a subject’s first ambulatory care visit for treatment of HZ was assigned as the index date for the purposes of this study. In order to limit our study sample to the adult population, this study included only subjects ≥18 years old. Ultimately, 47,359 cases were included in this study.

Similarly, we selected controls from the LHID2000. We first excluded all subjects who had a history of HZ since initiation of the NHI program in 1995. Thereafter, 142,077 subjects (three controls per case) were randomly selected and matched with cases by gender, age group (<45, 45∼64, and ≥65 years), and index year. While for cases, the year of the index date was the year in which the cases received their first diagnosis of HZ, for controls the year of the index date was simply a matched year in which controls had a medical utilization. We then assigned the date of their first use of medical services occurring during the index year as the index date for controls.

### Exposure assessment

The LHID2000 also includes ambulatory care medical orders, and this allowed us to identify subjects who filled prescriptions for statins prior to the index date. In this study, simvastatin, lovastatin, atorvastatin, fluvastatin, pravastatin, and rosuvastatin were selected as the major drugs of interest. We also defined subjects who had received continuous statin prescriptions for ≥60 days within 6 months before the index date as regular statin users. All other subjects who had been prescribed statin within 6 months before the index date were defined as irregular statin users.

### Statistical analysis

We used the SAS system (SAS System for Windows, vers. 8.2, SAS Institute, Cary, NC) for data analysis in this study. χ^2^ tests were performed to compare difference in the Charlson Comorbidities Index (CCI) score between cases and controls. The CCI was used to quantify preexisting comorbidities as a means of adjusting for the higher mortality risks associated with 19 medical conditions (congestive heart failure, myocardial infarction, liver disease, cancer, dementia, etc.). We performed conditional logistic regression analyses (conditioned on gender, age group, and index year) to calculate the odds ratio (OR) and its corresponding 95% confidence interval (CI) to present the association between HZ and having been previously prescribed statin. We used the conventional *p*≤0.05 for statistical significance in this study.

## Results

Of the 189,436 sampled subjects, the mean age was 51.7 (±17.8) years; the mean age among cases and controls was 51.2 and 51.9 years, respectively (*p* = 0.072). After matching for gender and age group, it shows that cases were more likely to have a CCO score of ≥4 than controls.

Table 1 presents the prevalences and ORs of statin users prior to the index date between cases and controls. In total, 13.0% of the sampled subjects had used statins before the index date, at 15.5% and 12.1% for cases and controls, respectively (*p*<0.001). In addition, the conditional logistic regression analysis (conditioned on gender, age group, and index year) suggested that the OR of being a statin user before the index date for cases was 1.28 (95% CI: 1.24∼1.32; *p*<0.001) compared to controls after adjusting for the CCI score.

**Table 1:** Prevalence, odds ratios (ORs), and 95% confidence intervals (CIs) for statin use among sampled subjects.

Variable	Total *(n* = 189,436)		Subjects with herpes zoster (*n* = 47,359)		Controls (*n* = 142,077)	
	*n*, %		*n*, %		*n*, %	
Statin use (including regular andirregular statin use)	24,523	13.0	7,315	15.5	17,208	12.1
Crude OR (95% CI)	–		1.33*** (1.29∼1.37)		1.00	
Adjusted OR (95% CI)	–		1.28*** (1.24∼1.32)		1.00	
Regular statin use	17,916	9.8	5370	11.8	12,546	9.1
Crude OR (95% CI)	–		1.34*** (1.29∼1.38)		1.00	
Adjusted OR (95% CI)	–		1.29*** (1.25∼1.34)		1.00	
Irregular statin use	6607	3.9	1945	4.6	4662	3.6
Crude OR (95% CI)	–		1.30***(1.23∼1.37)		1.00	
Adjusted OR (95% CI)	–		1.25*** (1.19∼1.32)		1.00	

Notes: The OR was calculated by a conditional logistic regression which was conditioned on age group, gender, and index year.

***p<0.001.

Adjustments were made for subject’s Charlson Comorbidity Index score.

Table 1 further shows the OR of regular and irregular statin use before the index date among sampled subjects. After adjusting for monthly income, geographic location, and the CCI score, the conditional logistic regression analysis revealed that the adjusted ORs of being regular and irregular statin users before the index date for cases were 1.29 (95% CI: 1.25∼1.34; *p*<0.001) and 1.25 (95% CI: 1.19∼1.32), respectively, compared to controls.

Table 2 presents the ORs of statin use before the index date according to age group. It shows that HZ was consistently and significantly associated with statin use across all age groups. It is noteworthy that subjects aged 18∼44 years had the highest adjusted ORs for prior statin use among cases compared to controls (OR: 1.69; 95% CI: 1.45∼1.92; *p*<0.001).

**Table 2:** Odds ratios (ORs) and 95% confidence intervals (CIs) for statin use among subjects with herpes zoster and controls by age group.

Use of statin	Age group (years)					
	18∼44		45∼64		>64	
	Subjects with herpeszoster *n*, %	Controls*n*, %	Subjects with herpeszoster *n*, %	Controls*n*, %	Subjects with herpeszoster *n*, %	Controls*n*, %
Yes	410 (2.7)	705 (1.5)	3679 (19.1)	7899 (13.7)	3226 (25.2)	8604 (22.6)
Crude OR (95% CI)	1.74*** (1.58∼2.02)	1.00	1.49*** (1.42∼1.55)	1.00	1.15*** (1.10∼1.21)	1.00
Adjusted OR (95% CI)	1.69*** (1.45∼1.92)	1.00	1.44*** (1.38∼1.50)	1.00	1.14*** (1.09∼1.20)	1.00

Notes: The OR was calculated by a conditional logistic regression which was conditioned on gender and index year.

***p<0.001.

Adjustments are made for subject’s Charlson Comorbidity Index score.

Furthermore, Table 3 shows the ORs of statin use before the index date according to gender. It indicates that HZ was consistently and significantly associated with statin use regardless of gender (adjusted OR: 1.32 for females and 1.23 for males).

**Table 3:** Odds ratios (ORs) and 95% confidence intervals (CIs) for statin use among subjects with herpes zoster and controls by gender.

Use of statin	Gender			
	Male		Female	
	Subjects with herpeszoster *n*, %	Controls *n*, %	Subjects with herpeszoster *n*, %	Controls *n*, %
Yes	3051 (13.9)	7380 (11.1)	4264 (16.8)	9828 (13.0)
Crude OR (95% CI)	1.28*** (1.23∼1.34)	1.00	1.36*** (1.31∼1.41)	1.00
Adjusted OR (95% CI)	1.23*** (1.18∼1.29)	1.00	1.32*** (1.27∼1.38)	1.00

Notes: The OR was calculated by a conditional logistic regression which was conditioned on age group and index year.

***p<0.001.

Adjustments were made for subject’s Charlson Comorbidity Index score.

## Discussion

We found that prior statin use was significantly associated with HZ occurrence across all age and gender groups in the present study. In general, statin users may increase the OR of HZ occurrence by about 1.3-times compared to non-statin users. Compared to previous studies, our result is very similar to the Canadian population-based study done (HR: 1.13) by Antoniou et al [8]. As a commonly prescribed medication, the association of statins and HZ should not be overlooked. The potential burden of HZ should be considered when statin is used in clinical settings. A previous study suggested using a retrospective observational study to investigate the effects of statin would encounter a “healthy-user bias”, which assumes that subjects using statin may have more health-promoting behaviors such as exercise, cigarette cessation, and an adequate diet program. This effect was considered one of the major confounding factors when studying the association between statin and its beneficial effects in outcomes of infectious diseases [11]. However, this hypothesis is not likely to explain the results of the present study since we found that statin use was significantly associated with an increased frequency and risk of HZ occurrence.

We found that prior statin use was significantly associated with HZ occurrence in a large-scale population case-control study. Although the mechanism of the finding is still unknown, physicians should be aware of this association when they prescribe statin in clinical settings. Further study is advised to confirm our findings and explore the underlying pathomechanism.

Several immunomodulatory effects were reported for statin. Statin can decrease proinflammatory cytokines such as tumor necrosis factor-α, interleukin (IL)-1β, IL-6, and IL-8 [12], [13]. Statin is associated with decreased T-cell activation by antigen-presenting cells and with regulating T-cell apoptosis [14]. Furthermore, statin may decrease chemotactic effects of neutrophils [15]. Therefore, statin can cause significant immune perturbation. Statin was proposed to have potential benefits in treating rheumatoid arthritis (RA) [3], [16]. Interestingly, an observational study suggested the use of disease-modifying anti-rheumatic drugs appeared to increase the risk of HZ in patients with RA [17]–[19]. This observation may support our findings of an association between statin use and HZ occurrence.

Interestingly, we found that younger statin users were at higher risk of suffering from HZ than their older counterparts in the present study. We hypothesized that this phenomenon may be partially explained by the characteristics of HZ occurrence. The prevalence of HZ increases in the elderly [20]. Therefore, the effects of statin on HZ may be diluted in the elderly.

We found the even irregular statin users were associated with an increased risk of HZ occurrence, although the risk for HZ occurrence in irregular statin users was lower than that of regular statin users. We proposed two hypothesis regarding these findings. First, the association between statin and HZ occurrence could be dose-dependent. A higher dose of statin may be associated with a higher risk of HZ occurrence. However, in the study by Antoniou et al., no difference of risk for HZ was demonstrated among the users with difference doses of statin [8]. The authors proposed this finding may be associated with limited power from only few people had increased their dose form baseline dose in study population. Second, the vulnerability of subjects using statins to HZ may be quite high. In such cases, subjects with little exposure under irregular statin use were still at a significantly higher risk of HZ occurrence compared to non-statin users.

There are some insufficiencies in present study. First, potential mis-coding and non-coding of health registry-based studies may hamper the results of the present study. Nevertheless, the Taiwan NHI Bureau has a cross-checking mechanism to facilitate the accuracy of the coding in this system. In general, it is believed that the LHID2000 has good quality for epidemiological analyses. Second, we pooled all types of statin in the analysis. Because the potency for immune-modulation may vary among different types of statin, specific types of statin may carry higher risks for HZ. Third, some variables regarding blood levels of lipid profile were lacking in the present study. Therefore, the status of dyslipidemia after being controlled by statin could not be determined. Potential confounding by underlying dyslipidemia of HZ occurrence still cannot be excluded in the present study. Fourth, some basic profiles of body weight, physical activity, nutritional status, and alcohol consumption which could act as potential confounding factors were not shown in the study. Fifth, although we adjusted for comorbidities and basic demographic data of subjects in the analysis, medication profiles were not adjusted to avoid excessively complex interactions in the regression model. Some medications such as corticosteroids and immunosuppressants may also be associated with HZ occurrence [21].

## References

[1] McFarlane SI, Muniyappa R, Francisco R, Sowers JR (2002) Clinical review 145: Pleiotropic effects of statins: lipid reduction and beyond. J Clin Endocrinol Metab 87: 1451–1458. 11932263 10.1210/jcem.87.4.8412

[2] Mihos CG, Salas MJ, Santana O (2010) The pleiotropic effects of the hydroxy-methyl-glutaryl-CoA reductase inhibitors in cardiovascular disease: a comprehensive review. Cardiol Rev 18: 298–304. 20926939 10.1097/CRD.0b013e3181f52a7f

[3] Mihos CG, Artola RT, Santana O (2012) The pleiotropic effects of the hydroxy-methyl-glutaryl-CoA reductase inhibitors in rheumatologic disorders: a comprehensive review. Rheumatol Int 32: 287–294. 21805349 10.1007/s00296-011-2008-6

[4] Yawn BP, Itzler RF, Wollan PC, Pellissier JM, Sy LS, et al (2009) Health care utilization and cost burden of herpes zoster in a community population. Mayo Clin Proc 84: 787–794. 19720776 10.4065/84.9.787 PMC2735428

[5] Stein AN, Britt H, Harrison C, Conway EL, Cunningham A, et al (2009) Herpes zoster burden of illness and health care resource utilisation in the Australian population aged 50 years and older. Vaccine 27: 520–529. 19027048 10.1016/j.vaccine.2008.11.012

[6] Weaver BA (2007) The burden of herpes zoster and postherpetic neuralgia in the United States. J Am Osteopath Assoc 107: S2–S7. 17488884

[7] Terao M, Yamamoto T, Umeda J (2005) Drug Induced Herpes Zoster (Do Statin induce Herpes Zoster?) Skin Res 4;335–338.

[8] Antoniou T, Zheng H, Singh S, Juurlink DN, Mamdani MM, et al (2014) Statins and the risk of herpes zoster: a population-based cohort study. Clinical Infectious Diseases 58: 350–356. 24235264 10.1093/cid/cit745 PMC3954107

[9] Kang JH, Chen YH, Lin HC (2010) Comorbidity profiles among patients with ankylosing spondylitis: a nationwide population-based study. Ann Rheum Dis 69: 1165–1168. 20375121 10.1136/ard.2009.116178

[10] Cheng CL, Kao YH, Lin SJ, Lee CH, Lai ML (2011) Validation of the National Health Insurance Research Database with ischemic stroke cases in Taiwan. Pharmacoepidemiol Drug Saf 20: 236–242. 21351304 10.1002/pds.2087

[11] Yende S, Milbrandt EB, Kellum JA, Kong L, Delude RL, et al (2011) Understanding the potential role of statins in pneumonia and sepsis. Crit Care Med 39: 1871–1878. 21516038 10.1097/CCM.0b013e31821b8290 PMC3139804

[12] Rosenson RS, Tangney CC, Casey LC (1999) Inhibition of proinflammatory cytokine production by pravastatin. Lancet 353: 983–984. 10.1016/S0140-6736(98)05917-0 10459915

[13] Rezaie-Majd A, Maca T, Bucek RA, Valent P, Müller MR, et al (2002) Simvastatin reduces expression of cytokines interleukin-6, interleukin-8, and monocyte chemoattractant protein-1 in circulating monocytes from hypercholesterolemic patients. Arterioscler Thromb Vasc Biol 22: 1194–1199. 12117737 10.1161/01.atv.0000022694.16328.cc

[14] Hakamada-Taguchi R, Uehara Y, Kuribayashi K, Numabe A, et al (2003) Inhibition of hydroxymethylglutaryl-coenzyme a reductase reduces Th1 development and promotes Th2 development. Circ Res 93: 948–956. 14563711 10.1161/01.RES.0000101298.76864.14

[15] Kaneider NC, Reinisch CM, Dunzendorfer S, Meierhofer C, Djanani A, et al (2001) Induction of apoptosis and inhibition of migration of inflammatory and vascular wall cells by cerivastatin. Atherosclerosis 158: 23–33. 11500171 10.1016/s0021-9150(00)00764-4

[16] Leung BP, Sattar N, Crilly A, Prach M, McCarey DW, et al (2003) A novel anti-inflammatory role for simvastatin in inflammatory arthritis. J Immunol 170: 1524–1530. 12538717 10.4049/jimmunol.170.3.1524

[17] Che H, Lukas C, Morel J, Combe B. Risk of herpes/herpes zoster during anti-tumor necrosis factor therapy in patients with rheumatoid arthritis. Systematic review and meta-analysis. Joint, bone, spine : revue du rhumatisme. (in press). 10.1016/j.jbspin.2013.07.009 23932722

[18] Wolfe F, Michaud K, Chakravarty EF (2006) Rates and predictors of herpes zoster in patients with rheumatoid arthritis and non-inflammatory musculoskeletal disorders. Rheumatology 45: 1370–1375. 17003175 10.1093/rheumatology/kel328

[19] Bongartz T, Orenstein R (2009) Therapy: The risk of herpes zoster: another cost of anti-TNF therapy? Nature reviews. Rheumatology 5: 361–S363. 19568250 10.1038/nrrheum.2009.120

[20] Schmader K, Gnann JW Jr, Watson CP (2008) The epidemiological, clinical, and pathological rationale for the herpes zoster vaccine. J Infect Dis 197: S207–S215. 18419399 10.1086/522152

[21] Whitley RJ, Gnann JW Jr (2009) Herpes zoster in the age of focused immunosuppressive therapy. JAMA 301: 774–775. 19224757 10.1001/jama.2009.150

